# Seawater Immersion Aggravates Burn Injury Causing Severe Blood Coagulation Dysfunction

**DOI:** 10.1155/2016/9471478

**Published:** 2016-01-18

**Authors:** Hong Yan, Qingxiang Mao, Yongda Ma, Li Wang, Xian Chen, Yi Hu, Hengjiang Ge

**Affiliations:** Department of Anesthesia, Daping Hospital and Research Institute of Field Surgery, Third Military Medical University, Chongqing 400042, China

## Abstract

This study aimed to investigate the endothelial function in a canine model of burn injury combined with seawater immersion. The model of burn injury was established. The dogs were randomly divided into four groups including dogs with burn injury (B group), or burn injury combined with seawater immersion (BI group), or only immersion in seawater (I group), or control animals with no injury or immersion (C group). The circulating endothelial cell (CEC) count and coagulation-fibrinolysis parameters were measured. The CEC count in B group increased at 4 h, 7 h, and 10 h after injury and then reduced, whereas it continuously increased to a greater extent in BI group (*P* < 0.05). The von Willebrand factor (vWF) activity, plasminogen activator inhibitor (PAI-1), and the ratio of thromboxane B2 (TXB_2_) to 6-keto-prostaglandin F1*α* (6-K-PGF_1*α*_) in BI group had a marked increase after injury, and the tissue-type plasminogen activator (tPA) in the BI group decreased. Microscope observations revealed thrombus formation in lungs of the animals in BI group, but not in C, I, or B groups. Burn injury causes endothelial dysfunction, and seawater immersion lastingly aggravates this injury, leading to a higher risk of developing thrombosis.

## 1. Introduction

Endothelial cells line the interior surface of blood vessels and lymphatic vessels of human body. They play a vital role in maintaining homeostasis by preventing thrombosis via the release of factors inhibiting coagulation and platelet adhesion and aggregation and those that lyse clots [1]. Events such as injury or inflammation cause disturbances of endothelial function, leading to the release of procoagulant factors, including fibronectin, von Willebrand factor (vWF), and platelet activating factor, and the establishment of a prothrombotic state [2]. Further, an injured endothelium attracts platelets, which subsequently adhere to the underlying extracellular matrix, resulting in thrombus formation [3].

Burn injury is a major cause of premature death or disability among military personnel or civilians involved in accidents, conflicts, and terrorist attacks worldwide. Previous studies have indicated that burn injury may cause endothelial barrier dysfunction [4], although the underlying mechanism is unclear. When such injuries are sustained at sea, the injured regions may be directly exposed to seawater in situations when the injured person is thrown overboard, for example, if the ship sinks or is severely damaged [5]. Seawater, which has alkaline pH, is a hypertonic solution with respect to the human body. On immersion of lesions of burn injury into seawater, the damaged vessels are exposed to a hypertonic environment and seawater infiltrates the damaged blood vessels. It is possible that immersion in seawater has a systemic impact on blood coagulation and fibrinolysis associated with endothelial dysfunction in animals with burn injury.

In the past, some reports have been published on the pathological changes associated with burn injuries [6, 7]; however, data on the changes associated with burn injury combined with seawater immersion are limited. In this study, we sought to investigate the effect of seawater exposure on the anticoagulant and fibrinolytic functions of the endothelium and the resultant changes in blood coagulation by using a canine model of burn injury exposed to seawater.

## 2. Materials and Methods

### 2.1. Study Protocol

The effects of seawater immersion on the endothelium were investigated by inducing burn injury in healthy adult dogs (Beagles, *N* = 20; age, 18–24 months; weight, 13.4 ± 1.6 kg). The animals were procured from the Institute of Laboratory Animal Science of Fujian Province. Approval of the Ethics Committee of the Third Military Medical University was sought before the start of the study. All the animal experiments were conducted in accordance with the stipulations of the Guide for the Care and Use of Laboratory Animals [8].

### 2.2. Preparation of Experimental Animal Models

Under general anesthesia induced by the intravenous administration of propofol (2 mg/kg), the dogs were smeared with 3% gelatinized gasoline on the back and limbs, and then the gasoline was ignited for 10 sec to induce a second-degree burn injury covering 10% of the total body surface area (TBSA), and the injury was confirmed by biopsy. For maintenance of anesthesia, intravenous infusion of buprenorphine (0.1 mg/kg, Sigma, St. Louis, MO, USA) was continued throughout the experiment.

Exposure of the burn injury to seawater was achieved by immersing the dog in the water (temperature, 21°C; salinity, 25.3‰; pH value, 8.1; Fujian Region, China) for 4 h, such that the head, neck, and forelimbs were out of the water.

### 2.3. Study Groups

The dogs were randomized into four groups with different experimental conditions: group B included dogs with burn injury (*n* = 6); group BI with burn injury followed by immersion in seawater (*n* = 6); group I with only immersion in seawater (*n* = 4); and group C without injury or immersion (the control group, *n* = 4).

### 2.4. Parameters and Measurements

Blood samples were collected from all dogs at six different time points (before injury and 4 h, 7 h, 10 h, 20 h, and 28 h after injury). The following parameters were then evaluated using the samples: the circulating endothelial cell (CEC) count and levels of vWF activity, tissue-type plasminogen activator (tPA), plasminogen activator inhibitor (PAI-1), thromboxane B2 (TXB_2_), and 6-keto-prostaglandin F_1*α*_ (6-K-PGF_1*α*_). To measure the CEC count, endothelial cells were isolated from 3 mL of the blood sample, and the cells were counted on a hemocytometer under an Olympus microscope to determine the number of CECs in the entire chamber (9 large squares). Since the volume in the chamber is 0.9 *μ*L, the total number of cells was divided by 9 to obtain the number of cells per microliter. The mean of three measurements was taken as the final measurement. The latex-immunoturbidimetric method was used to assess the level of vWF activity. The vWF activity assay kit (Shanghai Sun Biological Technology, China) was used according to the instructions provided by the manufacturer. Briefly, latex microparticles coated with specific vWF antibodies coagulated in the presence of vWF antigens, resulting in an increase in the turbidity that is directly proportional to the vWF concentration, which was then determined photometrically. Further, the levels of tPA and PAI-1 were examined using the chromogenic substrate method. The assay kits for tPA and PAI-1 (Shanghai Sun Biological Technology, China) were used as per the manufacturer's instructions. In short, tPA converts plasminogen into plasmin, which in turn cleaves the chromogenic substrate (S-2251) to form p-nitroaniline (pNA). The activity level of tPA was calculated in terms of the absorbance of pNA at 405 nm, which is directly proportional to the enzymatic activity of tPA. The PAI-1 level was determined as the amount of tPA required to neutralize PAI-1. Furthermore, a radioimmunoassay was performed to measure the levels of TXB_2_ and 6-K-PGF_1*α*_ by using an automatic gamma counter at the Radioimmunological Center of the Technology Development of PLA General Hospital. Besides the measurement of the above-mentioned parameters, histological analysis of the lung tissues was also performed. At the end of the 28-hour observation period, all the dogs were sacrificed by bloodletting. Lung tissue samples that were formalin-fixed, paraffin-embedded (5 *μ*m thickness), and stained with hematoxylin and eosin (HE) were used to evaluate the histological changes in the lungs.

### 2.5. Statistical Analysis

All statistical analysis was performed using the SPSS software package (version 16.0; SPSS Inc., Chicago, IL, USA). The quantitative data are presented as mean ± standard deviation (SD). The intergroup differences were analyzed by parametric or nonparametric ANOVA, and intragroup differences were determined using the *t*-test. The methods have been described in the paper. Statistical significance was set at a *P* value of <0.05.

## 3. Results

### 3.1. CEC Count

**Figure 1:**
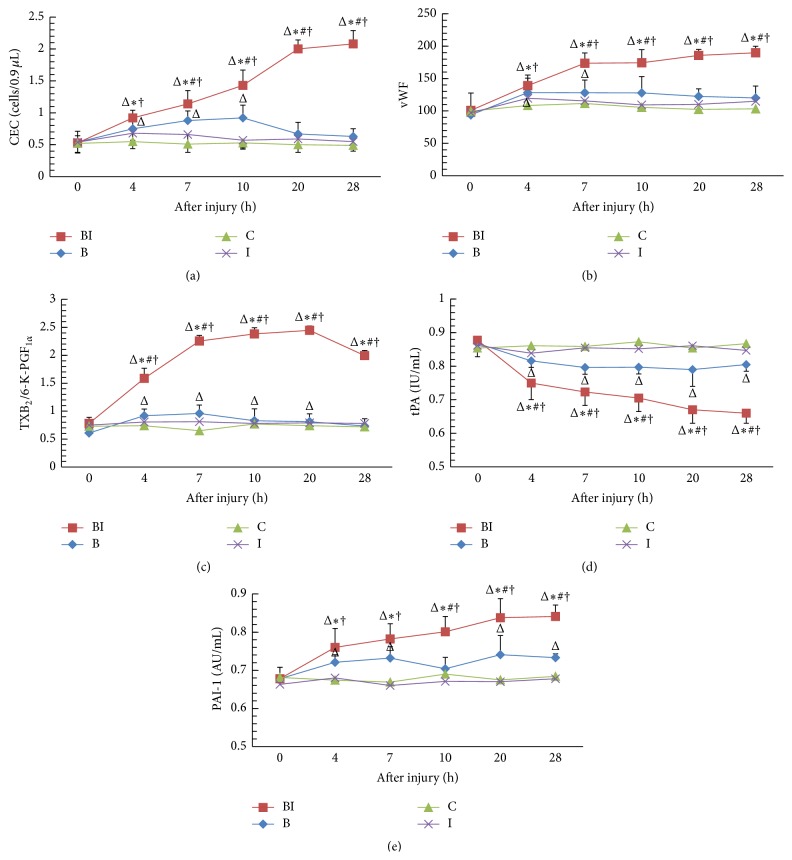
Analysis of the endothelial and coagulation-fibrinolysis function in the dogs of the four groups (B, BI, C, and I) after the injury (*n* = 20 in all) in terms of various factors: (a) circulating endothelial cell (CEC) count; (b) vWF activity; (c) the ratio of thromboxane B2 (TXB_2_) and 6-keto-prostaglandin F1*α* (6-K-PGF_1*α*_) levels; (d) the tissue-type plasminogen activator (tPA) level; and (e) the plasminogen activator inhibitor (PAI-1). ^#^ *P* < 0.05, compared to group B; ^*∗*^ *P* < 0.05, compared to group C; ^†^ *P* < 0.05, compared to group I, ^Δ^ *P* < 0.05, compared to before injury (0 h).

In groups C and I, no significant differences were noted between the pre- and postinjury values of the CEC count (*P* > 0.05). In group B, the CEC count increased during the first 10 h after injury to levels significantly higher than that before the injury (*P* < 0.05 at 4 h, 7 h, and 10 h after injury), after which it started decreasing. However, in group BI, the CEC count increased continuously and remained significantly higher than that in the other groups, at 7 h, 10 h, 20 h, and 28 h after injury (*P* < 0.05, Figure 1(a)).

### 3.2. vWF Activity

The postinjury levels of vWF activity in groups C and I did not differ from those before injury (*P* > 0.05). In group B, the levels of vWF activity at 4 h and 7 h after injury were higher than that before injury (*P* < 0.05). Further, in group BI, the vWF activity showed a marked increase at 7 h, 10 h, 20 h, and 28 h after injury and remained significantly higher than those in groups C, I, and B (*P* < 0.05, Figure 1(b)).

### 3.3. The Ratio of TXB_2_ to 6-K-PGF_1*α*_ (TXB_2_/6-K-PGF_1*α*_)

In groups C and I, no significant differences were noted between the pre- and postinjury values of the ratio of TXB_2_ to 6-K-PGF_1*α*_ (*P* > 0.05). In group BI, the value of the TXB_2_/6-K-PGF_1*α*_ ratio showed a continuous increase after injury, reaching the peak value at 20 h after injury and decreased thereafter; the value differed significantly from those in the other groups at all time points after injury (*P* < 0.05 at all time point after injury). However, the TXB_2_/6-K-PGF_1*α*_ ratio in group B showed an initial increase until 7 h after injury and then decreased gradually thereafter; the ratio remained higher than before injury (*P* < 0.05 at 4 h, 7 h, 10 h, and 20 h after injury; Figure 1(c)).

### 3.4. tPA and PAI-1

The postinjury levels of tPA and PAI-1 in groups C and I did not differ from those recorded before the injury (*P* > 0.05). Further, in groups B and BI, the tPA level decreased over time, with the decrease in group BI being greater than that in group B (*P* < 0.05) at all the postinjury time points (Figure 1(d)). In contrast, the PAI-1 levels in groups B and BI showed a tendency to increase with time after injury. Interestingly, the increase in the PAI-1 level in group BI was greater than that in group B. Further, the tPA and PAI-1 levels in groups B and BI differed significantly from those in groups C and I, at 10 h, 20 h, and 28 h after injury (*P* < 0.05; Figure 1(e)).

### 3.5. Histological Findings in Lung

**Figure 2:**
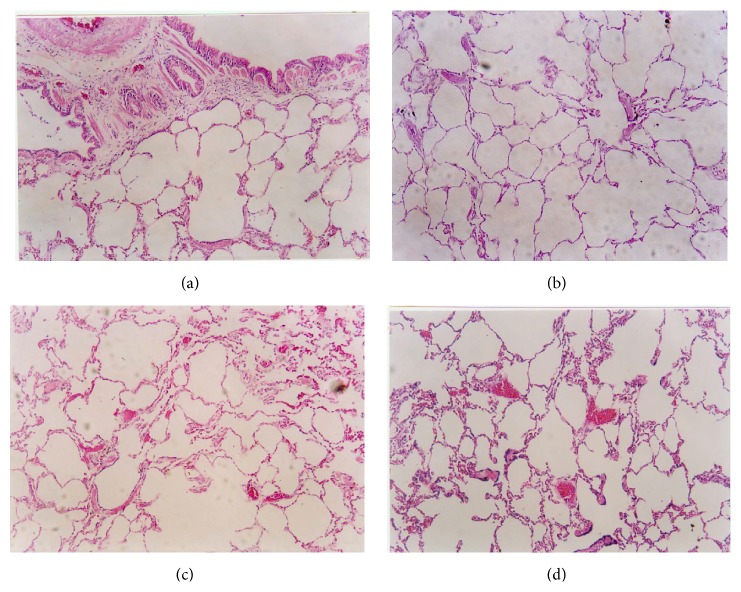
Histological findings of the lung tissue samples obtained for the different groups (magnification, 100x). (a) Normal lung tissue of the dogs in group C; (b) normal lung tissue of the dogs in group I; (c) absence of signs of thrombosis in lungs of the dogs and only lung capillary congestion, dilation, and blood stasis in the samples obtained from group B; (d) at 28 hours after injury, thrombus formation in lungs of the dogs in group BI.

Specimens of the lung tissues obtained from dogs in groups C, I, and B showed no signs of thrombosis or pulmonary edema; however, those obtained from the dogs in group BI showed evidence of thrombus formation and interstitial infiltration of red blood cells and inflammatory cells at 28 h after injury (Figure 2).

## 4. Discussion

The endothelium, which is formed by endothelial cells, acts as an antithrombotic surface that facilitates the transport of cellular constituents throughout the vascular system. In addition, the endothelium regulates blood flow by the synthesis and release of important antiplatelet and anticoagulant factors, such as PGI_2_, tPA, thrombomodulin (TM), antithrombin III (AT-III), and heparin [9]. However, certain internal and external pathogenic factors can damage the endothelium, whereby it loses its antithrombotic property and becomes prothrombotic instead; this results in thrombosis [10]. In this study, we investigated the effect of seawater immersion of burn injury and found that this combined insult causes severe endothelial dysfunction and disturbance of the coagulation-fibrinolytic balance in the canine model. Thus, the results of this study represent experimental evidence that highlights the importance of the appropriate clinical treatment of coagulation disorders caused by this type of injury.

One of the important pathological changes occurring in cases of burn injury is damage to the microvascular endothelium. The structure of the vessel wall can be disrupted by a multitude of mechanical factors, including the heat from the burn, thereby damaging the vascular endothelial cells [11]. In addition, most cases of burn injury are complicated by secondary infection. Therefore, several proinflammatory factors (i.e., cytokines and endotoxin) are produced and released at the infected site after the injury; this leads to the activation of endothelial cells and their damage. In addition, inflammatory mediators can trigger intracellular signaling cascades that can disrupt the endothelial barrier and increase microvascular endothelial cell hyperpermeability [12, 13]. Another cause of endothelial dysfunction is oxidative stress, which occurs as a systemic response after burn or trauma [14]. Reactive oxygen species produced due to oxidative stress lead to endothelial dysfunction as well as premature senescence or apoptosis of endothelial cells. Antioxidants have been shown to improve the endothelial function and clinical outcome in cases of burn injury [14]. CECs are endothelial cells that detach from the vascular wall and enter the peripheral circulation in response to endothelial injury; therefore, the quantification of CECs is considered a reliable method for assessing endothelial damage [15]. Our findings showed that burn injury led to an increase in the CEC count, thereby indicating the occurrence of acute vascular endothelial cell injury. Additionally, we also noted a reverse of the increase in CEC count at 10 hours after injury in group B. This implies that, in the absence of immersion, the animals had recovered gradually from the burn injury, probably due to a compensatory mechanism. However, group BI showed a continuous increase in the CEC count, which indicates that seawater immersion induced a more serious, lasting injury on the vascular endothelial cells. This may be attributed to a few reasons. First, the infiltration of seawater through disrupted vessel walls could create a hypertonic environment and consequently damage the cell membrane of the vascular endothelial cells [16]. Second, seawater immersion could trigger an inflammatory response, thereby aggravating endothelial damage, as mentioned above. Finally, investigations have shown that burn injury along with seawater immersion leads to the development of metabolic acidosis (our unpublished data) and the resultant production of ketones, lactic acid, and so forth, which cause direct damage to the endothelial cells. Thus, our findings showed that burn injury leads to damage of the primary vascular endothelial cells and that this damage is further aggravated by seawater immersion.

vWF is a highly prothrombotic blood glycoprotein that is stored within the Weibel-Palade bodies of endothelial cells. It promotes the adhesion of platelets to the sites of vascular injury and is therefore critical to maintaining hemostasis and thrombosis [17]. In addition, vWF serves as a chaperone for coagulation factor VIII in the plasma and protects it from degradation until it is delivered to the platelet surface, where coagulation factor VIII participates indirectly in blood coagulation [18, 19]. Since damage to endothelial cells leads to an increase in the level of circulating vWF, the latter is a suitable marker of endothelial dysfunction [20]. Studies have proven that an elevated circulating level of vWF is a risk factor for arterial and venous thrombosis [21, 22]. In the present study, group BI showed an elevated level of vWF activity after injury, thereby proving the occurrence of endothelial dysfunction in cases of burn injury combined with seawater immersion.

The fibrinolytic system is regulated by tPA and its physiological inhibitor PAI-1. tPA is serine protease produced by endothelial cells and mainly stored within; only a very small amount of tPA is present in the blood [23]. tPA plays a critical role in fibrinolysis by catalyzing the conversion of plasminogen to plasmin and thereby facilitating thrombus dissolution. Similar to tPA, PAI-1 is also mainly produced by the endothelial cells, and only a small portion of the total blood pool is present in the plasma; approximately 90% of the total blood pool of PAI-1 is stored in the alpha granules of the platelets [24]. PAI-1 is released in the event of vascular injury and is involved in maintaining the stability of fibrin clot. PAI-1 binds rapidly with tPA at a ratio of 1 : 1 to form a stable tPA/PAI-1 complex, which prevents the conversion of plasminogen to plasmin and thereby inhibits fibrinolysis [25]. Increased levels of plasma PAI-1 and decreased levels of tPA are risk factors for thrombosis and embolism [26]. In this study, both groups B and BI showed a decrease in the level of tPA and increase in the level of PAI-1 over time. However, the decrease or increase in the levels of tPA and PAI in group BI was greater than that in group B (*P* < 0.05); this implies that the disruption of the coagulation-fibrinolysis system in group BI is greater than that in group B.

The levels of TXB_2_ and 6-K-PGF_1*α*_ are often measured to assess the plasma levels of thromboxane A2 (TXA_2_) and PGI_2_, respectively [27]. TXA_2_ activates platelet aggregation, whereas PGI_2_ inhibits TXA_2_-induced platelet activation and aggregation [28]. Therefore, a balance between the plasma levels of TXA_2_ and PGI_2_ is important for effectively modulating platelet and vascular wall function and thereby maintaining homeostasis* in vivo*. An increase in the TXA_2_/PGI_2_ (TXB_2_/6-K-PGF_1*α*_) ratio is indicative of thrombosis. In our study, group BI showed a continued increase in the TXB_2_/6-K-PGF_1*α*_ ratio, thereby reflecting the imbalance between the levels of TXA_2_ and PGI_2_. Thus, we can infer that seawater immersion in the presence of burn injury increases the risk of developing thrombosis. This was confirmed by histological examination, which showed signs of thrombus formation and interstitial infiltration of red blood cells and inflammatory cells in the lung tissue samples obtained from group BI, whereas none of the other groups showed any such signs.

Our study has some limitations. Seawater has alkaline pH, and it is a hypertonic, nonsterile solution with relative low temperature with respect to the human body. This study investigated the effect of seawater on blood coagulation dysfunction after bury injury, but which factor(s) above and how to cause aggravation were not clear. Further investigations are needed to improve our understanding of the pathological changes in such cases.

In conclusion, our findings indicate that seawater immersion aggravates the endothelial dysfunction occurring after burn injury and increases the risk of thrombosis. An insight into the key pathological changes in such cases would provide a theoretical basis for the development of successful management strategies for such patients to facilitate the timely administration of antibiotics, achieve effective improvement in microcirculation, and prevent the development of a hypercoagulable state. Our findings also indicate the importance of using hemostatic agents with caution in such patients.
